# Supplementary material for: Highly-efficient electrically-driven localized surface plasmon source enabled by resonant inelastic electron tunneling
Source: Nat Commun. 2021 May 25;12:3111. doi: 10.1038/s41467-021-23512-2 (PMC8149681; doi:10.1038/s41467-021-23512-2)
Supplement: Supplementary file 1 — Supplementary information [file 41467_2021_23512_MOESM1_ESM.pdf]

Highly-efficient electrically-driven localized surface plasmon source enabled by resonant inelastic electron tunneling

Haoliang Qian, Shilong Li, Su-Wen Hsu, Ching-Fu Chen, Fanglin Tian, Andrea R. Tao and Zhaowei Liu

Content

Note 1. Device fabrication

Note 1.1 Fabrication of resonant tunneling junctions

Note 1.2 Synthesis of silver nanorods

Note 1.3 Assembly and transfer of large-scale silver nanorods array

Note 2. Calculation of electron transport

Note 2.1 Electron resonant states in metallic quantum wells

Note 2.2 Resonant inelastic electron tunneling in metallic quantum wells

Note 3. Simulations of optical response

Note 3.1 Optical emission simulation

Note 3.2 Calculation of surface plasmon radiation efficiency

Note 3.3 Optical scattering simulation

Note 4. Electrical and optical measurements

Note 4.1 Equivalent circuit

Note 4.2 Electrical and optical powers

Note 4.3 System detection efficiency

Note 4.4 Far-field emission spectra

Note 4.5 Discussion on emission powers

Note 5. Calculation of external quantum efficiency

Note 6. Second derivative of *I–V* curves

Note 6.1 Comparison with IET sources

Note 6.2 Analysis of RIET samples

Note 6.3 Analysis of control samples

Note 7. Discussion on plasmonic circuitry integration

Note 1. Device fabrication

Note 1.1 Fabrication of resonant tunneling junctions

For the resonant inelastic electron tunneling (RIET) devices, as schematically shown in Fig. 2A, both the 50-nm TiN (the bottom electrode) and the above metallic quantum well (MQW) junction were grown on sapphire substrates by the reactive magnetron sputtering technique (AJA International) [1]. The reactive growth temperature of TiN was set as 350 °C with a N_2:Ar gas ratio of 7:3. The Ti target was used with a power of 200 W. The Al_2O_3 was deposited in the same chamber as the TiN reactive growth; its deposition ambiance is given as follows: an Al_2O_3 target was used; the temperature for the Al_2O_3 deposition was set as 350 °C—the same as that for the reactive growth of TiN; the deposition power was set as 150 W with 5-sccm Ar under 5-mT pressure; the deposition speed was about 0.4 nm/min. The cross-sectional morphology of the MQW junction films was characterized by high-resolution transmission electron microscopy (HRTEM), as shown in Figs. 1C and 1D. The top electrode, from bottom to top, is composed of 60-nm Al_2O_3 (magnetron sputtering technique, AJA International), 5-nm Ti and 150-nm Au (Temescal BJD 1800). The different working regions were produced by the photolithography (Heidelberg MLA150). The silver nanorods (AgNRs) array was transferred from the water-air interface described below. In the end, the 150-nm ITO was deposited (magnetron sputtering technique, AJA International) using a 100-W deposition power with 5-sccm Ar under 5-mT pressure.

Note 1.2 Synthesis of silver nanorods

The AgNRs were synthesized by modifying a previously reported seed-mediated synthesis of faceted nanorods [2, 3]. First, silver nanocrystal seeds were made by using a mixture of 1.500 mL of 0.05 M sodium citrate, 0.045 mL of 0.05 M PVP (molecular weight ~ 55000), 0.150 mL of 0.005 M l-arginine, 0.600 mL of 0.005 M $AgNO_3$ and 18.000 mL of deionized water in a 20-

mL vial with a magnetic stirring. Then, the reducing agent 0.24 mL of 0.10 M $NaBH_4$ was added. The resulting solution became bright yellow after few minutes. This bright yellow solution was then exposed to a blue LED lamp (Thorlabs M455L4). After exposed about 20 hours, the resulting solution became bright yellowish orange with a plasmonic peak at 450 nm.

In seed growth step, 6 mL of the freshly prepared seed solution was centrifuged and re-dispersed in 1.0 mL of deionized water. 12.0 mL of deionized water, 1.6 mL of 0.05 M sodium citrate, and 0.264 mL of 0.05 M PVP (molecular weight ~ 55000) were heated to 100 °C in a 20-mL vial on a magnetic stirrer. After temperature equilibration, 1 mL of seed solution was added followed by 0.005 M silver nitrate. Varying the amount of silver nitrate (0.7–1.2 mL) and the reaction time (30–90 min) allowed producing rods of different length with an aspect ratio up to 3–4 in high yield, as seen from the ultraviolet/visible spectra and SEM images shown in Fig. S1.

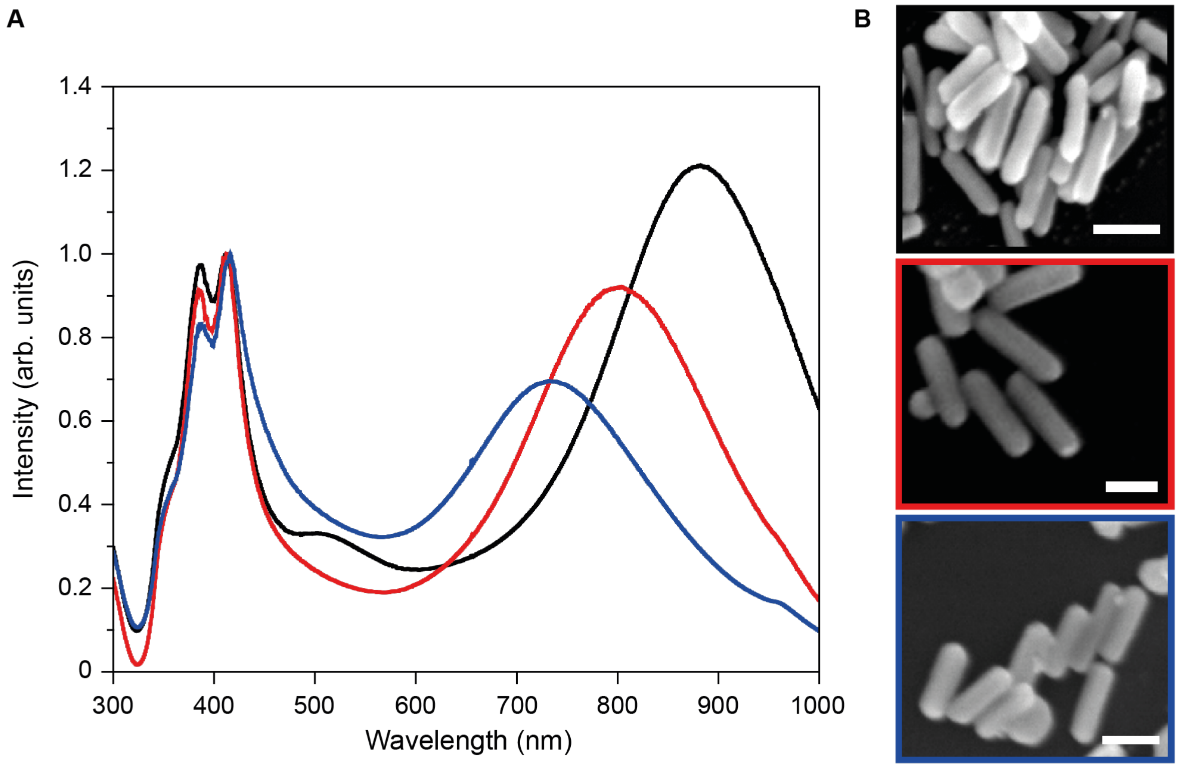

Fig. S1. Characterization of AgNRs. (**A** and **B**) Extinction spectra (A) of AgNR aqueous solution with different morphologies, and the respective SEM images (B). Scale bar is 100 nm.

Note 1.3 Assembly and transfer of large-scale silver nanorods array

To prepare the orderly nanocrystals array, the further purification and surface modification steps require for as-made AgNRs colloidal solution. A 3–4 ml of as-made AgNRs aqueous solution was centrifuged for removing small nanocrystals and free ligands (PVP and citrate). And then the sediment was re-dispersed in 0.75-ml deionized water and followed by adding 0.75-ml ethanol. This AgNRs water/ethanol solution was added into a 1-μM poly(ethylene glycol) methyl ether thiol (PEG-thiol with average Mn ~ 10000) ethanol solution (20 mg of PEG-thiol + 2-ml ethanol) and incubated for 2–3 hours. During the surface modification, original ligand shell (PVP and citrate) coated outside nanorods was replaced by PEG thiol. After 2–3 hours, the solution was centrifuged for removing the PVP, citrate and free PEG thiols. And then the sediment was re-dispersed ethanol and this purification process was repeated three times. The finial sediment was dispersed in $CHCl_3$. This colloidal nanocrystal solution was then added dropwise to the air-water interface of glass petri dish, which gives an isotopically distributed monolayer of silver nanocrystals floating at the air-water interface and the spacing between nanocrystals can be sophisticatedly controlled, as shown in Fig. S2. Nanocrystal monolayers were then transferred onto the MQW junction.

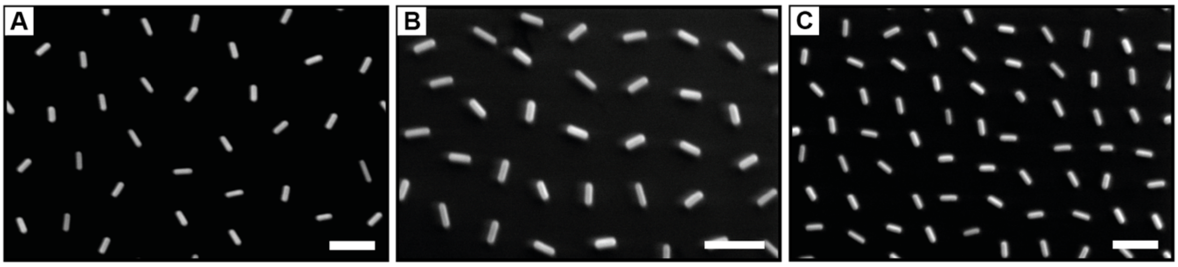

Fig. S2. Characterization of AgNRs array. (**A** to **C**) SEM images showing PEG-thiol coated AgNRs arrays with different spacing on Si substrate, which were transferred from the monolayer of AgNRs floating at the air-water interface. Scale bar is 500 nm.

Note 2. Calculation of electron transport

Note 2.1 Electron resonant states in metallic quantum wells

Owning to the quantum size effect, the conduction band of an ultrathin metal film is split into resonant electron subbands. To calculate these electron resonant states, quantum-mechanical simulations on the MQW junction structure shown in Fig. S3 were performed using the commercial software package COMSOL Multiphysics. Specifically, quasi bound states of this structure were searched by solving the one-dimensional (1D) time-independent Schrödinger equation with an Open Boundary condition on both the two exterior boundaries (i.e. the two end points) of the modeling domain—i.e., wave functions leak out of the modeling domain without reflection back at the two end points. To match to the experimental observations, these parameters [4-8] were determined: $m_{\mathrm{eff,Al2O3}} = 1.0m_{\mathrm{e}}$, $\mathrm{EA}_{\mathrm{Al2O3}} = 1.4$ eV, $t_{\mathrm{Al2O3}} = 10$ nm, $m_{\mathrm{eff,ITO}} = 0.44m_{\mathrm{e}}$, $\mathrm{WF}_{\mathrm{ITO}} = 4.35$ eV, $\mathrm{EF}_{\mathrm{ITO}} = 0.535$ eV, $m_{\mathrm{eff,TiN}} = 1.1m_{\mathrm{e}}$, $\mathrm{WF}_{\mathrm{TiN}} = 4.65$ eV, $\mathrm{EF}_{\mathrm{TiN}} = 4.5$ eV, $t_{\mathrm{TiN}} = 1.4$ nm, and $\tau_{\mathrm{TiN}} = 10$ fs, where m_{eff}, EA, t, WF, EF, and τ represent, respectively, the electron effective mass, the electron affinity, the thickness, the working function, the Fermi level, and the resonant-state lifetime. It is worth noting that the effect of band bending and Schottky barrier was negligible because all the interfaces in the MQW junction consist of either oxide/metal (i.e. Al_2O_3/TiN) or oxide/conductive-oxide (i.e. Al_2O_3/ITO).

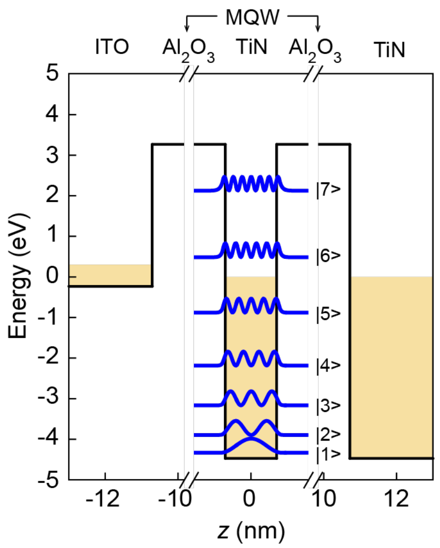

Fig. S3. Electronic subbands of the MQW in RIET devices. Wave functions (blue lines) are superimposed at the respective eigen energies. Fermi seas (light yellow fills) are superimposed up to the respective Fermi levels.

Note 2.2 Resonant inelastic electron tunneling in metallic quantum wells

The process of inelastic electron tunneling (IET) with the excitation of surface plasmons (SPs) can be described as a spontaneous emission process of an electron during its transition from a higher energy state in one electrode to a lower energy state in another electrode. The same is true for resonant inelastic electron tunneling (RIET) in an MQW junction, as demonstrated in this work. According to the Bardeen's transfer Hamiltonian formalism [9], by applying the Fermi's Golden Rule to the one-dimensional tunneling problem as defined in Fig. S4A, the inelastic tunneling rate in vacuum reads [10, 11]

$$\gamma_{\mathrm{inel}}^{0}(\nu, V_{\mathrm{b}}) = \frac{\pi e^2}{3h\nu m_{\mathrm{eff}}^2 \varepsilon_0} \rho_0 \int_{h\nu}^{eV_{\mathrm{b}}} |\mathcal{P}(\nu, E)|^2 \rho_{\mathrm{L}}(E) \rho_{\mathrm{R}}(E - h\nu) \mathrm{d}E, \tag{S1}$$

where ε_0 is the vacuum permittivity, ρ_{L} (ρ_{R}) is the electronic density of state of the left (right) electrode, while $\mathcal{P}$ is the matrix element for this tunneling process and given by

$$\mathcal{P}(\nu, E) = -i\hbar \int_{z_1}^{z_2} \Psi_{\mathrm{R}}^{*}(E - h\nu) \frac{\mathrm{d}}{\mathrm{d}z} \Psi_{\mathrm{L}}(E) \mathrm{d}z. \tag{S2}$$

The wave functions of the two electrodes Ψ_{L} and Ψ_{R} are obtained with the COMSOL package.

For the calculations of Ψ_L and Ψ_R, the 1D time-independent Schrödinger equation in two subsystems with the potentials shown, respectively, in Figs. S4B and S4C was solved. Unlike the quasi bound states calculation discussed above, where the wave function propagates outward on both open boundaries, here, the wave function propagates inward from one boundary and goes out of the other boundary for each subsystem; specifically, the incident wave function (i.e. Ψ_L) was normalized to the unit flux of probability, while the transmitted wave function (i.e. Ψ_R) was normalized to the unit density of probability [12]. It is worth noting that since there is no resonant elastic electron tunneling (REET) in the working voltage range (i.e. 1.6–2.8 V) of the RIET sources (Fig. 3), one can safely calculate Ψ_L with the subsystem shown in Fig. S4B, without including the MQW structure.

Figure S4D shows a typical vacuum inelastic tunneling rate spectrum. As mentioned in the main text, there is a cutoff frequency v_max that follows the quantum relation $hv_\mathrm{max} = eV_\mathrm{b}$. It is a signature of all IET-based sources [13]. For the RIET sources demonstrated here, there is another cutoff frequency v_co in this spectral range, above which the RIET process is not supported.

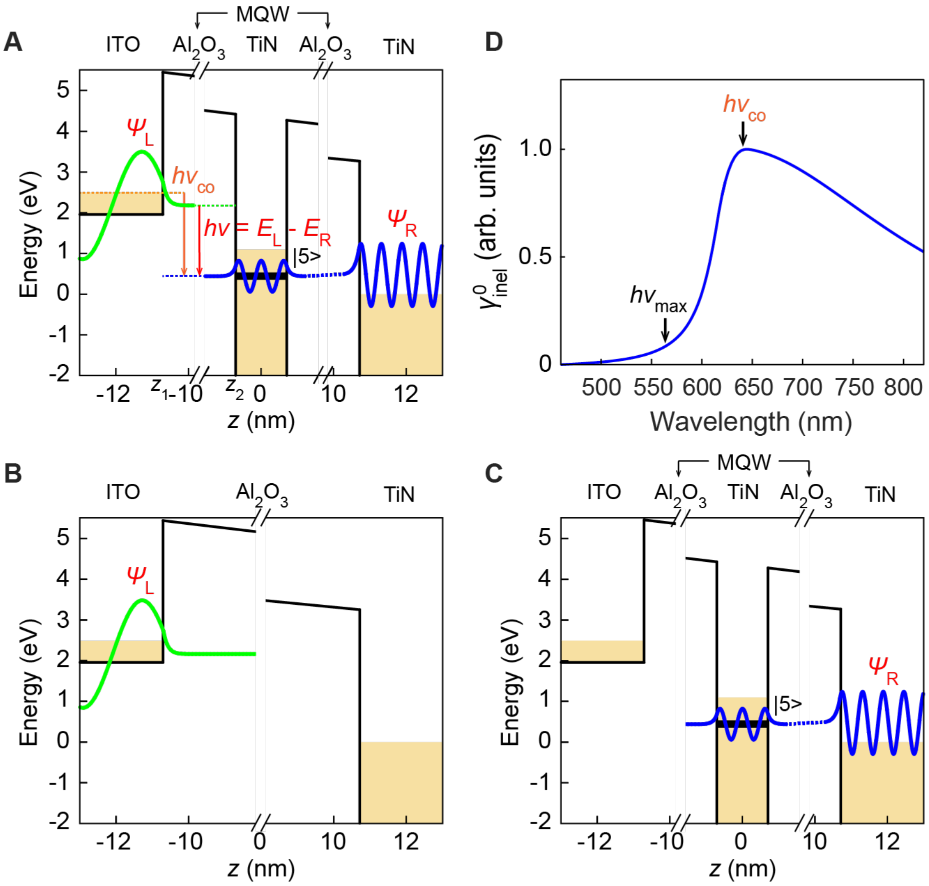

Fig. S4. Modeling of RIETs in the MQW. (**A**) Wave functions Ψ_{L} and Ψ_{R} used to calculate the vacuum inelastic tunneling rate $\gamma_{\mathrm{inel}}^{0}$. (**B** and **C**) Subsystems used to calculate the wave functions Ψ_{L} (B) and Ψ_{R} (C). The wave function Ψ_{R} in the middle TiN region is enlarged for clarity. (**D**) Typical $\gamma_{\mathrm{inel}}^{0}$ spectrum. A bias voltage of 2.2 V is applied.

Note 3. Simulations of optical response

Note 3.1 Optical emission simulation

To characterize the optical emission property for the RIET sources, the enhancement ($\rho_{\mathrm{opt}}/\rho_0$) of local density of optical state (LDOS), far-field radiation efficiency (η_{r}) and near-field SP radiation efficiency (η_{SP}) were simulated by solving the three-dimensional (3D) Maxwell's equations with the perfectly matched layer (PML) absorbing boundary condition using the COMSOL package. All geometries and materials were set to match the real sample conditions, while a dipole source was used and placed at the middle of the top Al_2O_3 barrier layer along the growth direction to mimic the inelastic tunneling emission process (Fig. S5J). The η_{r} was calculated as $\eta_r(\lambda) = p_{\mathrm{r}}/p_{\mathrm{tot}}$, where p_{tot} is the total power dissipation generated from the dipole source and p_{r} is the far-field power within the 0.5-NA collection angle (in order to keep the same as the experimental conditions). The η_{SP} was calculated as $\eta_{\mathrm{SP}}(\lambda) = p_{\mathrm{SP}}/p_{\mathrm{tot}}$, where the p_{sp} represents the SP power flow (see Section S3.2 for details). The polarization states (i.e. the x, y and z polarizations) of the dipole source and its lateral positions (i.e. y = 0, 20, and 40 nm) were varied respectively, and the results are summarized in Figs. S5A to S5I. The averaged $\eta_{\mathrm{r,avg}}$ was weighted by the LDOS as $\eta_{\mathrm{r,avg}} = \sum_i \eta_{\mathrm{r},i}\rho_{\mathrm{opt},i}/\sum_i \rho_{\mathrm{opt},i}$, where i represents the data obtained at the different polarization states and lateral positions; the result is shown in Fig. S5K. Figure S5L shows the LDOS-weighted average $\eta_{\mathrm{SP,avg}}$, i.e., $\eta_{\mathrm{SP,avg}} = \sum_i \eta_{\mathrm{SP},i}\rho_{\mathrm{opt},i}/\sum_i \rho_{\mathrm{opt},i}$. Note that the simulation results using more lateral positions (i.e. y = 0, 10, 20, 30, 40, and 50 nm) were used in Fig. 4.

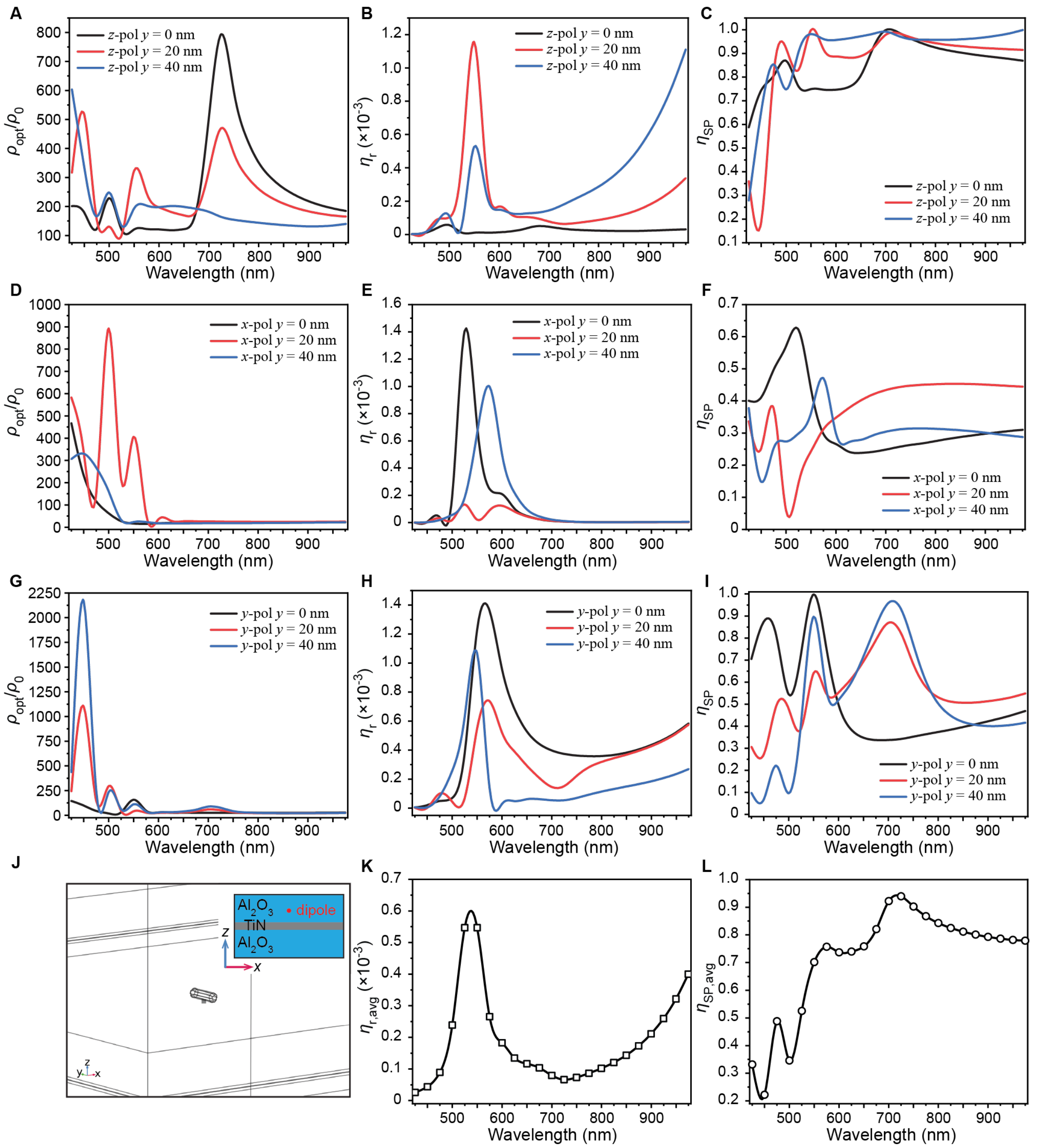

Fig. S5. Modeling of optical emission of the RIET SP sources. (**A** to **I**) Wavelength dependence of ρ_{opt}/ρ_0 (A, D, and G), η_{r} (B, E, and H), and η_{SP} (C, F, and I). (**J**) Snapshot of the geometry used in the simulations. The inset is a schematic drawing showing the position of the dipole source. (**K** and **L**) Wavelength dependence of the LDOS-weighted average $\eta_{\text{r,avg}}$ (K) and $\eta_{\text{SP,avg}}$ (L).

Note 3.2 Calculation of surface plasmon radiation efficiency

The procedure to calculate the surface plasmon radiation efficiency η_{SP} is shown in Fig. S6. Here, we took the η_{SP} at the wavelength of 740 nm for the z-polarized dipole source at the position $y = 0$ nm as the example, and all other dipole positions (i.e. $y = 20$ and 40 nm) with other dipole polarizations (i.e. x and y polarizations) were obtained using the same procedure.

As mentioned above, the η_{SP} was evaluated by $\eta_{SP} = p_{SP}/p_{tot}$, where p_{SP} represents the SP power flow and p_{tot} is the total power generated. As shown in the inset of Fig. S6B, a dipole source ($y = 0$ nm, z polarization) was set to be at the center of the AgNR in the xy-plane and 5 nm away from the bottom of the AgNR along the z-direction. Figure S6A shows the xz-plane cross-section through the dipole source, where the y coordinate represents the distance to this dipole. The SP power flow was calculated by the integration of the time-averaged Poynting vector with two xz-planes which have the equal distance to the dipole position (since the power flow will propagate on both sides from the dipole position) and the η_{SP} was thus obtained correspondingly. The calculated η_{SP} in different xz-planes (i.e. at different y-distances to the dipole) is shown in Fig. S6B. There was a power-flow fluctuation when the integration plane was very close to the dipole, and after some distances, the power flow became stabilized with certain plasmonic modes, showing an almost linearly decayed η_{SP}. Therefore, the η_{SP} for this dipole position ($y = 0$ nm) with this polarization state (z polarization) was obtained by the extrapolation of these linear-decay values.

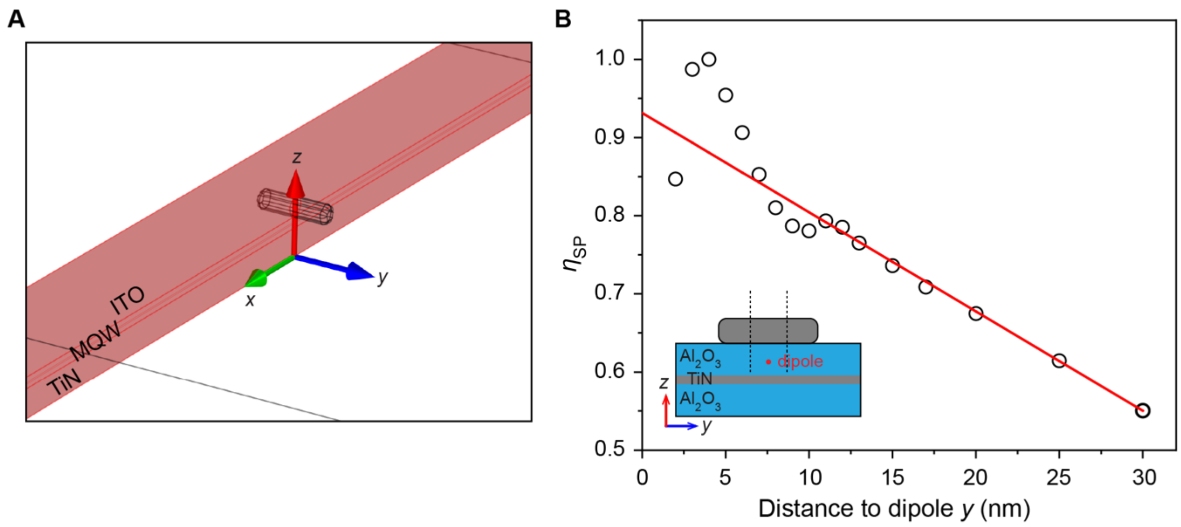

Fig. S6. Calculation of SP radiation efficiency. (**A**) Snapshot of the geometry used in the simulation showing the integration-plane for the SP power flow calculation. (**B**) Position dependence of the η_{SP}. Each data point was obtained by the integration of the time-averaged Poynting vector with two xz-planes that have the equal distance to the dipole position, as shown by the two dashed lines in the inset. The η_{SP} at this dipole position with this polarization state was calculated by the extrapolation of data in the linear-decay region (red line).

Note 3.3 Optical scattering simulation

Plane-wave sources were used in the far-field scattering simulations. Since the orientations of the AgNRs on top of the RIET sources are arbitrary, these plane-wave sources were set to have a 45° incident angle and an azimuthal angle of 45° with respect to the AgNR (Fig. S7A), for general reference purpose. Scattering spectra from the *p*- and *s*-polarized plane-wave sources were also averaged to match the dark-field scattering experimental conditions.

As shown in Fig. S7B, the experimental result was well fitted with these simulations. By analyzing the respective charge distribution, we can distinguish the different SP modes supported by the RIET sources, as follows: the mode-1 centered at ~550 nm is due to the longitudinal octupolar resonance, i.e. $N = 3$ SP mode, as shown in Fig. 2H. The mode-3

centered at ~500 nm and the mode-4 centered at ~1280 nm are, respectively, the $N = 4$ and $N = 1$ SP modes; since they have little contribution to the scattering in the spectral range that we are interested in, we do not show them in the main text. As shown in Fig. 2I, coupling between the $N = 2$ SP mode and the gap plasmon mode results in the mode-2 centered at ~740 nm.

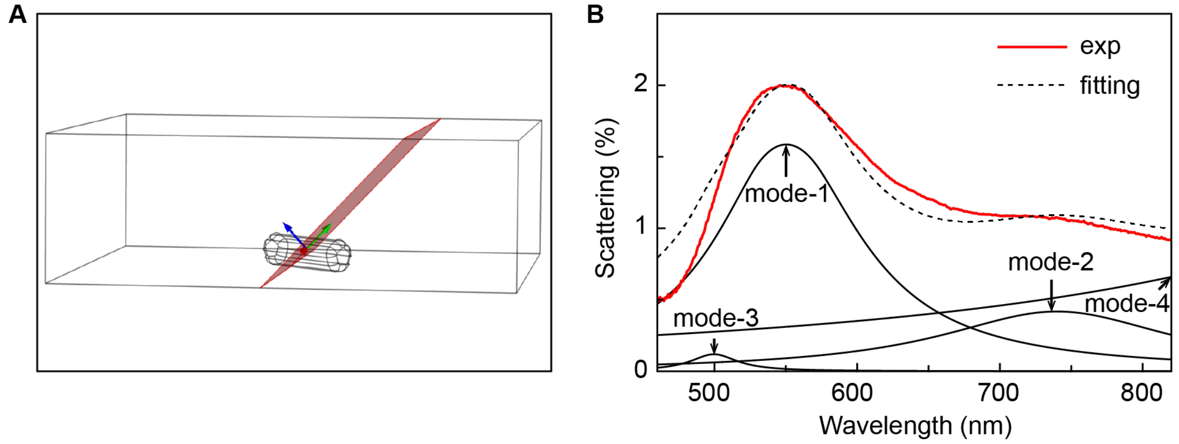

Fig. S7. Modeling of optical scattering of the RIET SP sources. (**A**) Snapshot of the geometry used in the simulation showing the orientation of the incident plane-wave (red plane) with respect to the AgNR. (**B**) Dark-field optical scattering spectrum (red solid line) with the simulation fitting (black dashed line). All four plasmonic resonance peaks are superimposed in the scattering spectrum.

Note 4. Electrical and optical measurements

Note 4.1 Equivalent circuit

As shown in Fig. S8, there are two conductive paths for a RIET SP source: One with ITO and another with ITO and AgNR; they are connected in parallel. Since the electrical resistance of AgNR covered with 1–2 nm organic insulating layer (i.e. PEG) is much larger than that of the ITO, the current will go along the conductive path without the AgNR.

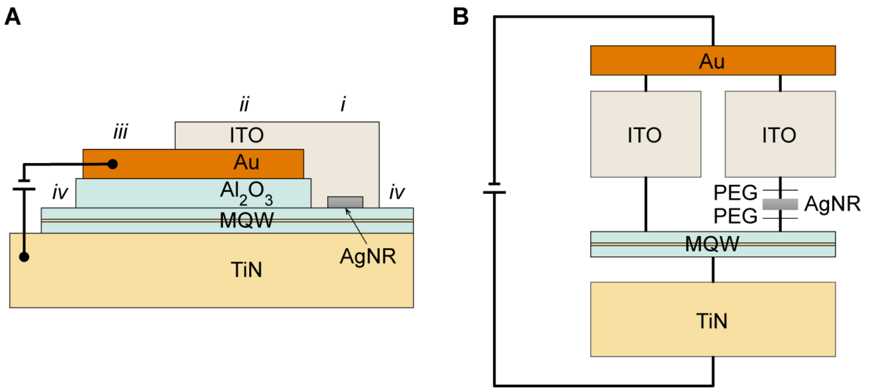

Fig. S8. Structural cross-section of a RIET SP source (A) and its equivalent circuit (B). The organic insulating layer (PEG) covering the AgNR is explicitly shown in (B). Figure A taken from Fig. 2a.

Note 4.2 Electrical and optical powers

To analyze the electro-optical response of the RIET sources, current–voltage (*I*–*V*) curves of a device and its optical emissions were recorded. The *I*–*V* measurements were done with the Agilent B1500 semiconductor device analyzer. The optical emission image was taken by an EMCCD (Andor iXon^{EM}+897). The optical emission spectra were obtained with the monochromator Shamrock 500i (Andor). In the voltage-dependent emission power measurements, the monochromator was set as a 30-nm bandwidth with a 30-nm increment step to ensuring that only the signal within the spectral range of [$\lambda - 15$ nm, $\lambda + 15$ nm] can be detected for each step. All the optical emission power measurements were done by a

photomultiplier tubes (PMT) from Hamamatsu Photonics (H10720-20). The total optical power emitted from the device was obtained by a spectral sum as $p_{\mathrm{tot}} = \sum \frac{p_{\mathrm{d}}(\lambda)}{\eta_{\mathrm{r,avg}}(\lambda) \times \eta_{\mathrm{SDE}}(\lambda)}$, where $p_{\mathrm{d}}(\lambda)$ is the detected power and $\eta_{\mathrm{SDE}}(\lambda)$ is the system detection efficiency (SDE). All the electronic and optical components were integrated with probe stations using a microscope objective of 50X with a 0.5 NA.

Note 4.3 System detection efficiency

As shown in Fig. S9A, the wavelength-dependent system detection efficiency $\eta_{\mathrm{SDE}}(\lambda)$ was measured by placing a tunable laser source (SuperK EXW-12, NKT Photonics, with a SuperK VARIA tunable single-line filter; the laser source NA is much smaller than the objective NA) with a known power $p_{\mathrm{i}}(\lambda)$ at the sample position, and then collecting the signal power $p_{\mathrm{d}}(\lambda)$, such that $\eta_{\mathrm{SDE}}(\lambda) = p_{\mathrm{d}}/p_{\mathrm{i}}$, and the results are shown in Fig. S9B. It is worth noting that the monochromator placed at the image plane has a F-number of f/6.5, where the collection NA is much larger than the optical signal at the image plane considering the 50X 0.5-NA objective; and the monochromator slit is much larger than the micro/nanojunction size on the image plane; therefore, there is no issue to couple all the light at the image plane into the monochromator.

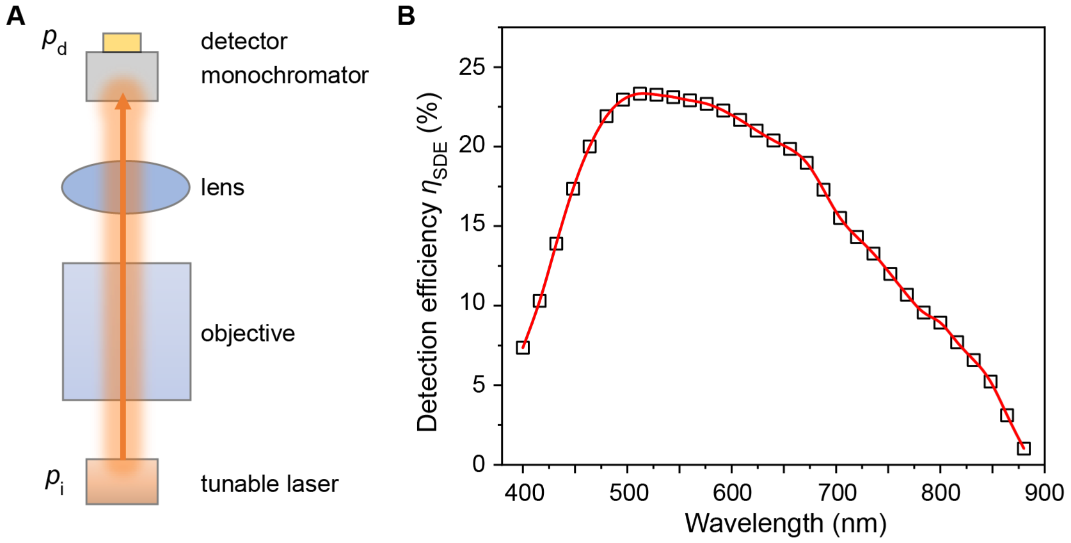

Fig. S9. Measurement of system detection efficiency. (**A**) Schematic drawing of the measurement system. The input power p_i is given, while the output power p_d is detected. (**B**) Wavelength-dependent system detection efficiency $\eta_{SDE} = p_d/p_i$ in the visible to near-infrared spectral range.

Note 4.4 Far-field emission spectra

The measured far-filed emission spectra S_r for the RIET device discussed in Fig. 4 are shown in Fig. S10. They were used to obtain the SP emission spectra S_{SP} via the $\eta_{r,avg}$ given in Fig. S5K, i.e. $S_{SP} = S_r/\eta_{r,avg}$, and the results are shown in Fig. 4A.

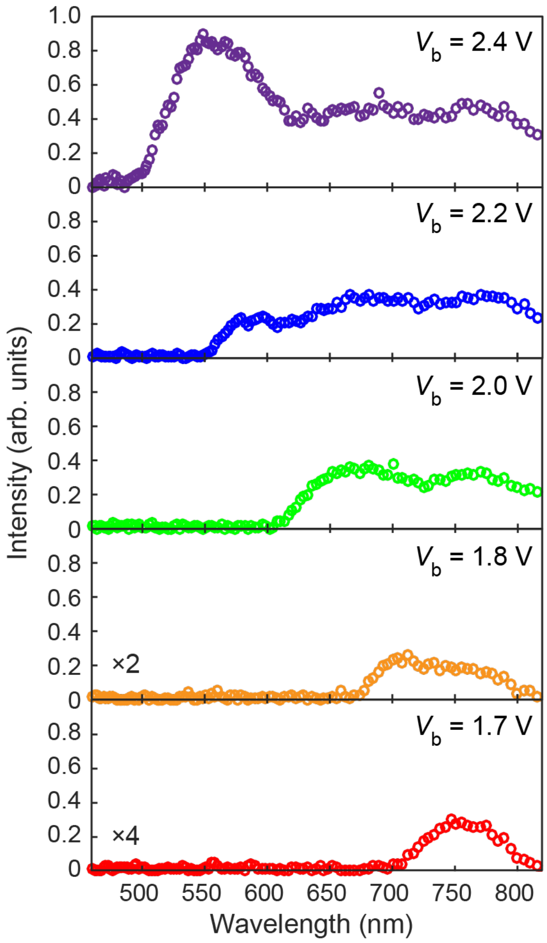

Fig. S10. Voltage dependence of the far-field emission spectrum for a RIET device. The wavelength-dependent system detection efficiency was taken into account.

Note 4.5 Discussion on emission powers

The emission power of the current RIET plasmon source is on the order of 1 nW; correcting for the far-field radiation efficiency of ~10^{-3}, the far-field emission power is on the order of 1 pW (8×12 μm^2 emission area), on par with that reported in a recent work[14] where an electrical surface plasmon source based on IET has a far-field emission power on the order of 10 pW (25×45 μm^2 emission area).

The emitted power from the RIET source is mainly limited by the thick tunneling barriers. Therefore, to brighten the RIET source one could reduce the barriers either by improving the

material quality of the Al_2O_3 barrier currently used in this work or by using other high-quality barrier materials such as 2D boron nitride stacked layers.

Note 5. Calculation of external quantum efficiency

The procedure to calculate the external quantum efficiency (EQE) of the RIET sources is shown in Fig. S11. Here, we took the 30% EQE obtained at the voltage of 1.7 V as the example, and all the other EQEs were calculated using the same procedure.

The power detected $p_{\mathrm{d}}(\lambda)$ is shown in Fig. S11A, where each data represents the total power within the spectral range of [$\lambda - 15$ nm, $\lambda + 15$ nm]. The system detection efficiency $\eta_{\mathrm{SDE}}(\lambda)$ is shown in Fig. S11B, while the entire efficiency curve is given in Fig. S9B. The simulated far-field radiation efficiency $\eta_{\mathrm{r,avg}}(\lambda)$ is shown in the left side of Fig. S11C, which is the ratio between the far-field emission power within the 0.5-NA collection angle to the total power generated from the dipole source inside the junction area (Section S3.1). The right side of Fig. S11C shows the simulated SP radiation efficiency $\eta_{\mathrm{SP,avg}}(\lambda)$, representing the ratio between the plasmon source power to the total power (Section S3.1). So, the SP power from the RIET source is calculated as $p_{\mathrm{SP}}(\lambda) = \left((p_{\mathrm{d}}/\eta_{\mathrm{SDE}})/\eta_{\mathrm{r,avg}}\right) \times \eta_{\mathrm{SP,avg}}$, and the result is shown in Fig. S11D. Since the number of SPs is $N_{\mathrm{SP}}(\lambda) = p_{\mathrm{SP}}/(hc/\lambda)$, we have EPE $= \sum p_{\mathrm{SP}}(\lambda)/(I \times V)$ and EQE $= \sum N_{\mathrm{SP}}(\lambda)/I$, where EPE is the external power efficiency.

The EQE of the devices were deduced by combining the IV measurements, far-field optical measurements and the simulation results. The multiple simulation results were averaged to get a more accurate far-field efficiency $\eta_{\mathrm{SP,avg}}(\lambda)$ as described in Section S3.1. The geometrical parameters of the device for these simulations were obtained according to the SEM and TEM results. The uncertainty of the AgNRs' size was well estimated by fitting the scattering spectrum with the simulations (Section S3.3). The determination of the system detection efficiency $\eta_{\mathrm{SDE}}(\lambda)$ and the far-field optical measurements were fulfilled with each data range of [$\lambda - 15$ nm, $\lambda + 15$ nm] to avoid point-like fluctuations as described in Sections S4.2 and

S4.3. In the end, multiple IV measurements and far-field optical measurements were performed and the resultant error bars were produced and given in Fig. 3C.

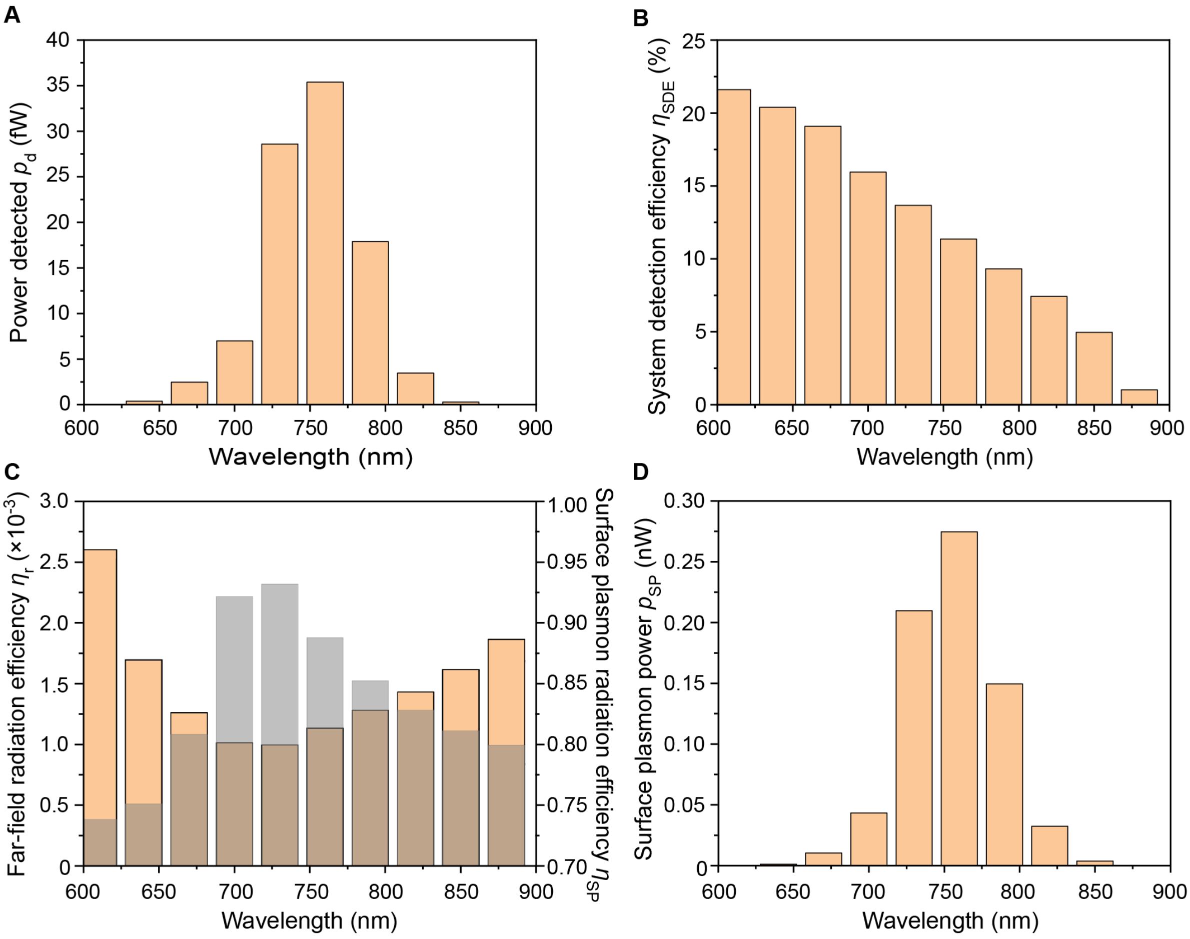

Fig. S11. Procedure to evaluate EQE. (**A** to **D**) Wavelength dependences of the power detected p_d (A), the system detection efficiency η_SDE (B), the far-field radiation efficiency $\eta_\mathrm{r,avg}$ (yellow, left side of C), the surface plasmon radiation efficiency $\eta_\mathrm{SP,avg}$ (gray, right side of C), and the surface plasmon power p_SP (D).

Note 6. Second derivative of *I–V* curves

Note 6.1 Comparison with IET sources

For a non-resonant IET junction, the second derivative $\mathrm{d}^2I/\mathrm{d}V^2$ of its *I–V* curve shows a peak at the voltage V_b satisfying the quantum relation $h\nu_{\max} = eV_\mathrm{b}$, as shown in Fig. S12A. It is thus a much more direct way of measuring the cutoff frequency $\nu_{\max}$ for IET junctions, compared to inspecting the turning point of the *I–V* curve. Figure S12B shows the $\mathrm{d}^2I/\mathrm{d}V^2$ curve for a RIET device, where a dip with a negative value is centered around the characteristic frequency defined mainly by the device's discrete electronic level. It is thus an alternative way of determining the characteristic frequency for RIET devices. As shown below, the second derivative provides us a very useful tool for understanding the electro-optical property of control samples, especially for those without RIET.

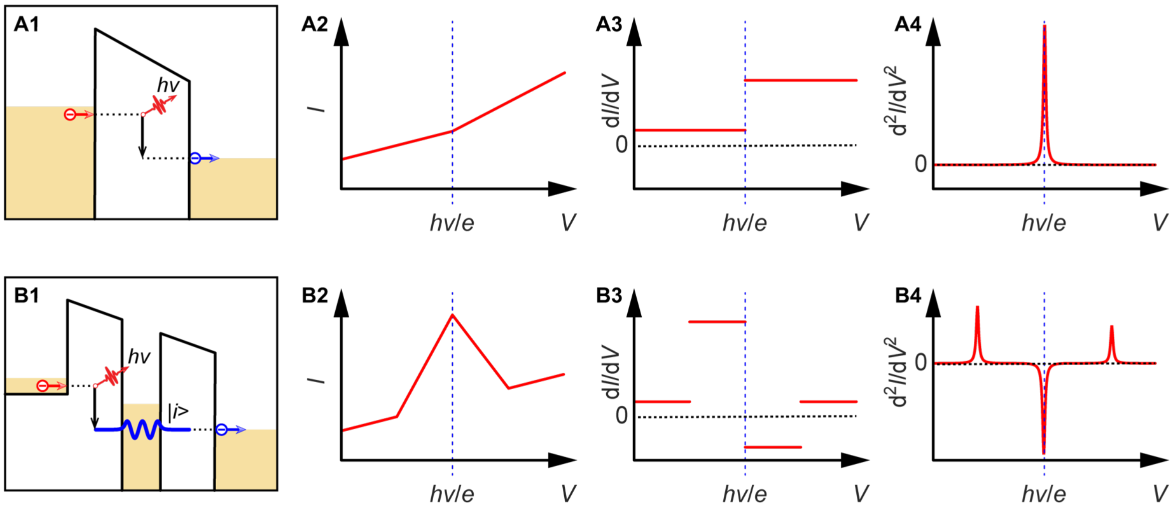

Fig. S12. Expected *I–V* curves and their first and second derivatives for non-resonant (A) and resonant (B) inelastic electron tunneling junctions, respectively. Figure A adapted from Ref. Science 319, 1056 (2008) [15].

Note 6.2 Analysis of RIET samples

Figure S13 shows the $\mathrm{d}^2I/\mathrm{d}V^2$ curves given in Fig. 3a. As expected from Fig. S12B, the $\mathrm{d}^2I/\mathrm{d}V^2$ curve for the RIET device shows two negative-valued dips centered, respectively, at the peaks 2 and 3 of the *I*–*V* curve. Therefore, it is a cross-check in determining the position of these current peaks.

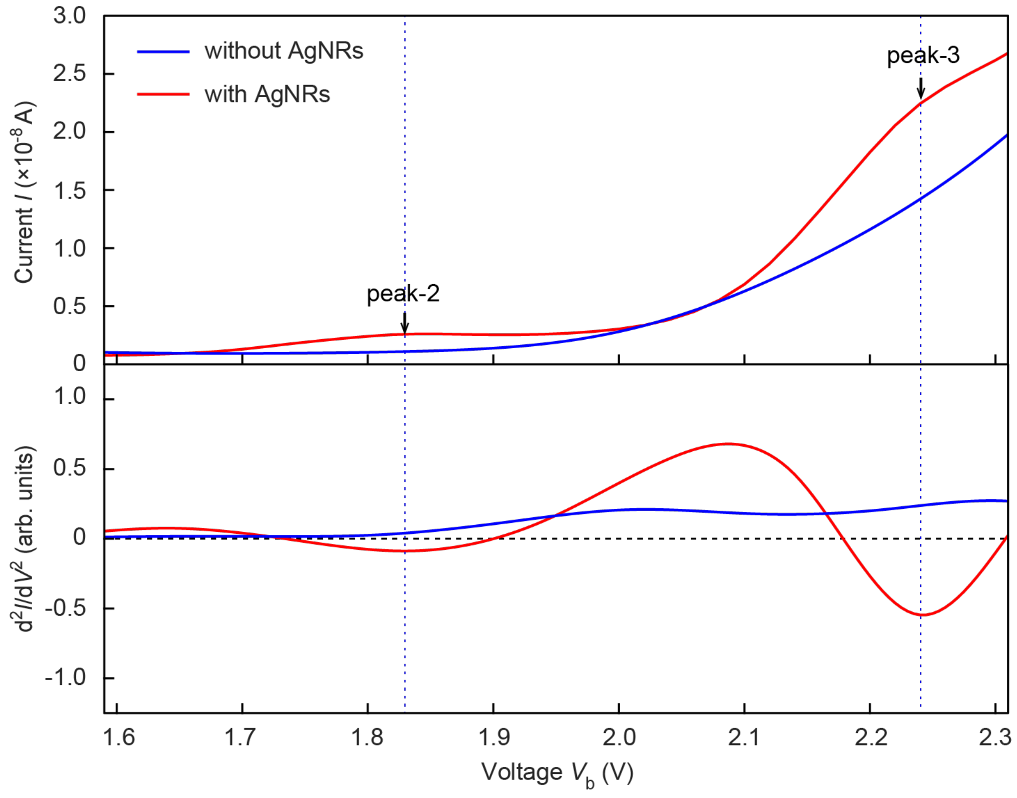

Fig. S13. Measured *I*–*V* curves and their respective second derivatives for a RIET device (with AgNRs) and its control sample (without AgNRs). Data taken from Fig. 3a.

Note 6.3 Analysis of control samples

Figure S14 shows the measured *I*–*V* curve and its $\mathrm{d}^2I/\mathrm{d}V^2$ for the control sample in the absence of MQWs; according to Fig. S12, there is no RIET but an IET at the voltage of ~2.5 V. The emission signals from this control sample were within the noise level. We attribute these results to the following reasons: This control sample actually constitutes an IET junction

where surface plasmons are supported by either the TiN electrode or the AgNRs; however, owing to 1) the very thick barrier thickness of 20-nm Al_2O_3 (thus the resultant ultra-small current on the order of few hundreds fA) and 2) the low photon generation efficiency without any RIET enhancement, the possibility of IET is extremely small—only observable from the d^2I/dV^2 curve.

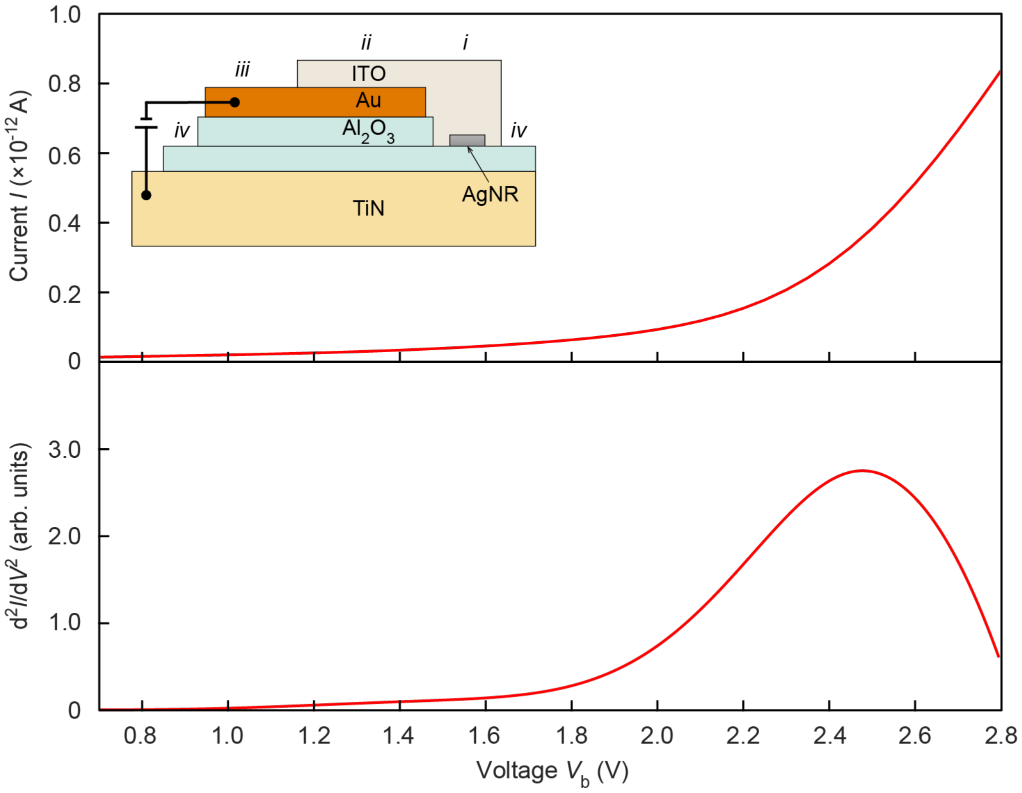

Fig. S14. Measured *I–V* curve and its second derivative for the control sample in the absence of MQWs.

Figure S15 shows the measured *I–V* curve and its d^2I/dV^2 for the RIET device with reversed bias; according to Fig. S12, it only shows the IET signature at the voltage of ~2.5 V. The emission signals under the revered-bias condition were within the noise level. We explain these results as follows. First of all, because of the large Fermi energy of the TiN electrode, the REET via the electronic states of the TiN QW coexists with the RIET in the reversed-bias condition;

as a result, the RIET efficiency is low—the resultant RIET current is thus too weak to be observed, even from the $\mathrm{d}^2I/\mathrm{d}V^2$ curve. Moreover, the initial and final states for an electron transport cover a wide range of the applied voltages due also to the large Fermi energy of the TiN electrode; thus, there is no signature for the REET current in the $\mathrm{d}^2I/\mathrm{d}V^2$ curve as well. Nevertheless, the RIET device in the reversed-bias condition constitutes an IET junction where surface plasmons are supported by either the TiN electrode or the AgNRs. However, the very thick barrier thickness of ~20-nm Al_2O_3 makes the observation of the IET event from the emission spectrum impossible.

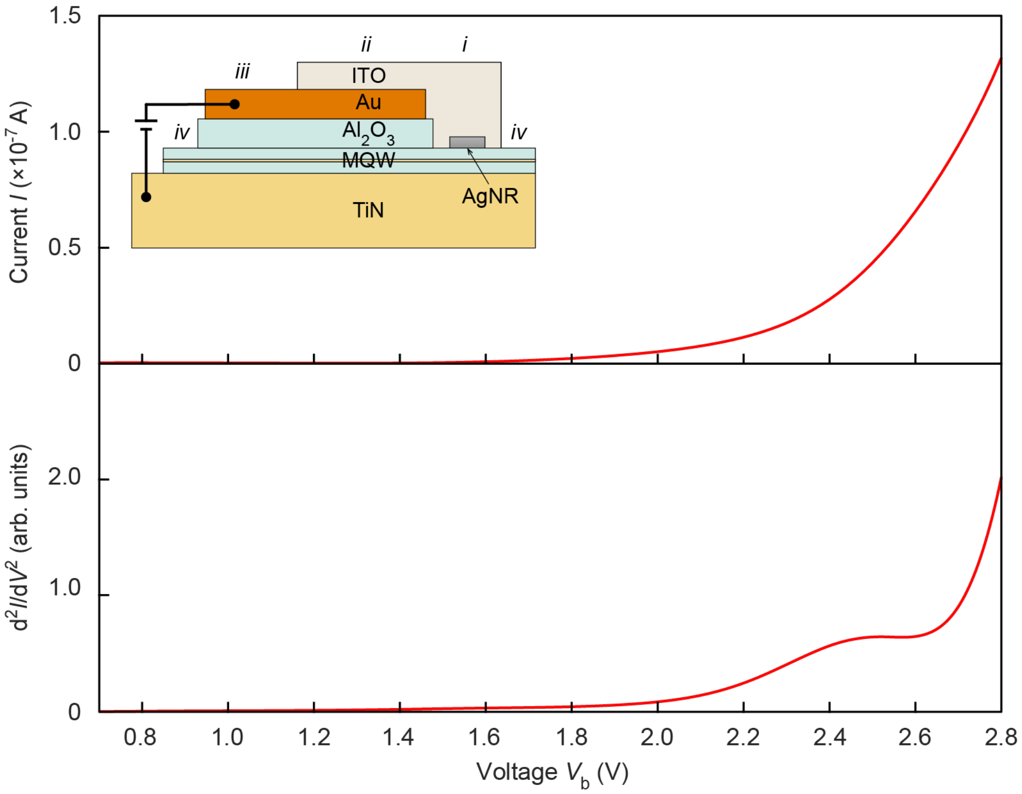

Fig. S15. Measured *I*–*V* curve and its second derivative for the RIET device with reversed bias.

Note 7. Discussion on plasmonic circuitry integration

Compared with silver nanocubes (AgNCs), AgNRs provide more degrees of freedom in the design of surface plasmon modes and their near-field excitation efficiency. It seems that these surface plasmons can hardly be used as they are localized plasmons in AgNRs which are embedded in ITO. However, as the source for a plasmonic circuitry, these AgNRs have to be integrated into the circuitry via a plasmonic waveguide, as shown in Fig. S16A, where a plasmonic slot waveguide is used to bridge the AgNRs and the plasmonic circuitry. By doing this, these surface plasmons can be used for optical sensing or information processing.

From the source integration point of view, the silver nanowires (AgNWs) can be used to replace both the AgNRs and the additional plasmonic waveguide, as shown in Fig. S16B, where the AgNWs themselves can be directly used to generate and then guide the surface plasmons into a plasmonic circuitry. However, the far-field efficiency of the surface plasmons from the AgNWs is too low to ensure a sufficient far-field measurement, so that they were not used in this work.

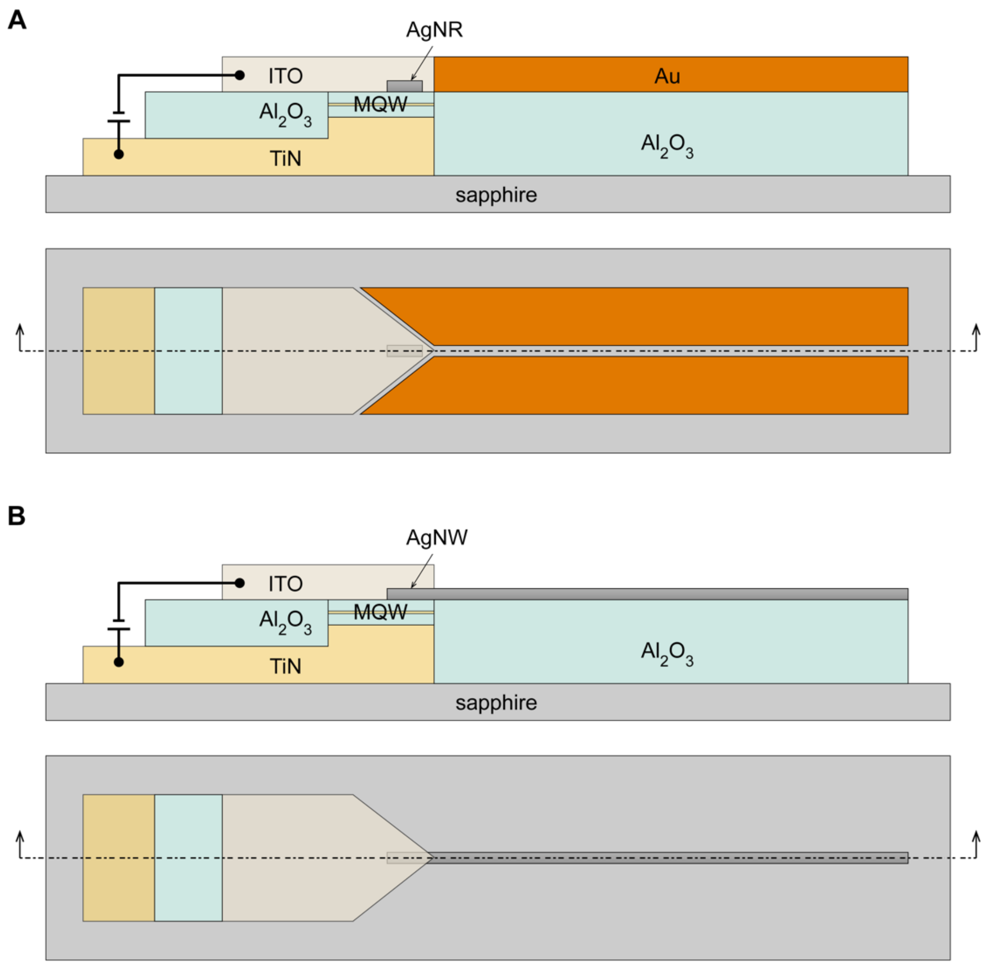

Fig. S16. Integration of MQW-based RIET surface plasmon sources into a plasmonic circuitry. (**A**) Schematic showing that a single AgNR can be used to support the surface plasmon excitation and an additional plasmonic waveguide, such as the slot waveguide, is needed for the transportation of the generated surface plasmon. (**B**) Schematic showing that a single AgNW can be used to support and transport the surface plasmon.

Supplementary References

1. Patsalas, P., Kalfagiannis, N., Kassavetis, S. Optical Properties and Plasmonic Performance of Titanium Nitride. *Materials* **8,** 3128-3154 (2015).

2. Zhang, J., Langille, M.R., Mirkin, C.A. Synthesis of Silver Nanorods by Low Energy Excitation of Spherical Plasmonic Seeds. *Nano Lett.* **11,** 2495-2498 (2011).

3. Pietrobon, B., McEachran, M., Kitaev, V. Synthesis of Size-Controlled Faceted Pentagonal Silver Nanorods with Tunable Plasmonic Properties and Self-Assembly of These Nanorods. *ACS Nano* **3,** 21-26 (2009).

4. Liu, X.*, et al.* Quantification and impact of nonparabolicity of the conduction band of indium tin oxide on its plasmonic properties. *Appl. Phys. Lett.* **105,** 181117 (2014).

5. Gassenbauer, Y., Klein, A. Electronic and Chemical Properties of Tin-Doped Indium Oxide (ITO) Surfaces and ITO/ZnPc Interfaces Studied In-situ by Photoelectron Spectroscopy. *The Journal of Physical Chemistry B* **110,** 4793-4801 (2006).

6. Shah, D.*, et al.* Optical Properties of Plasmonic Ultrathin TiN Films. *Advanced Optical Materials* **5,** 1700065 (2017).

7. Qian, H.*, et al.* Large optical nonlinearity enabled by coupled metallic quantum wells. *Light Sci. Appl* **8,** 13 (2019).

8. Alimardani, N.*, et al.* Investigation of the impact of insulator material on the performance of dissimilar electrode metal-insulator-metal diodes. *J. Appl. Phys.* **116,** 024508 (2014).

9. Bardeen, J. Tunnelling from a Many-Particle Point of View. *Phys. Rev. Lett.* **6,** 57-59 (1961).

10. Parzefall, M., Novotny, L. Optical antennas driven by quantum tunneling: a key issues review. *Rep. Prog. Phys* **82,** 112401 (2019).

11. Parzefall, M., Novotny, L. Light at the End of the Tunnel. *ACS Photonics* **5,** 4195-4202 (2018).

12. Uskov, A.V.*, et al.* Excitation of plasmonic nanoantennas by nonresonant and resonant electron tunnelling. *Nanoscale* **8,** 14573-14579 (2016).

13. Lambe, J., McCarthy, S.L. Light Emission from Inelastic Electron Tunneling. *Phys. Rev. Lett.* **37,** 923-925 (1976).

14. Zhang, C.*, et al.* Antenna surface plasmon emission by inelastic tunneling. *Nat. Commun.* **10,** 4949 (2019).

15. Galperin, M., Ratner, M.A., Nitzan, A., Troisi, A. Nuclear Coupling and Polarization in Molecular Transport Junctions: Beyond Tunneling to Function. *Science* **319,** 1056 (2008).
